# Sex differences but no evidence of quantitative honesty in the warning signals of six‐spot burnet moths (*Zygaena filipendulae* L.)*

**DOI:** 10.1111/evo.13505

**Published:** 2018-07-02

**Authors:** Emmanuelle Sophie Briolat, Mika Zagrobelny, Carl Erik Olsen, Jonathan D. Blount, Martin Stevens

**Affiliations:** ^1^ Centre for Ecology and Conservation, College of Life and Environmental Sciences University of Exeter Penryn Cornwall TR10 9FE United Kingdom; ^2^ Plant Biochemistry Laboratory and Copenhagen Plant Science Centre Department of Plant and Environmental Sciences University of Copenhagen 40 Thorvaldsensvej, DK‐1871 Frederiksberg C Copenhagen Denmark

**Keywords:** Aposematism, cyanogenic glucosides, honest signaling, *Zygaena*

## Abstract

The distinctive black and red wing pattern of six‐spot burnet moths (*Zygaena filipendulae*, L.) is a classic example of aposematism, advertising their potent cyanide‐based defences. While such warning signals provide a qualitatively honest signal of unprofitability, the evidence for quantitative honesty, whereby variation in visual traits could provide accurate estimates of individual toxicity, is more equivocal. Combining measures of cyanogenic glucoside content and wing color from the perspective of avian predators, we investigate the relationship between coloration and defences in *Z. filipendulae*, to test signal honesty both within and across populations. There were no significant relationships between mean cyanogenic glucoside concentration and metrics of wing coloration across populations in males, yet in females higher cyanogenic glucoside levels were associated with smaller and lighter red forewing markings. Trends within populations were similarly inconsistent with quantitative honesty, and persistent differences between the sexes were apparent: larger females, carrying a greater total cyanogenic glucoside load, displayed larger but less conspicuous markings than smaller males, according to several color metrics. The overall high aversiveness of cyanogenic glucosides and fluctuations in color and toxin levels during an individual's lifetime may contribute to these results, highlighting generally important reasons why signal honesty should not always be expected in aposematic species.

Warning coloration, or aposematism, is one of the key adaptive explanations for the bright and colorful patterns on show in the animal kingdom. Conspicuous visual signals act to warn potential predators that a prey item is toxic or otherwise unprofitable, a theory first proposed by Alfred Russell Wallace in relation to colorful caterpillars (Wallace 1867). Despite a long history of research into animal warning signals, many issues surrounding this topic remain unresolved, perhaps most notably the question of signal honesty in aposematism. While the evolution of qualitatively honest signals to predators, reliably indicating the presence of a defence, is inherent in the definition of aposematism and has strong support from both empirical work and theoretical modeling, evidence for quantitative honesty, in which the value of a signal reflects the level of the signaler's defences, is more mixed. Theoretical investigations into the potential for evolution and maintenance of honesty in warning signals have yielded conflicting predictions (reviewed in Summers et al. 2015). Similarly, empirical studies testing the relationship between properties of visual signals and toxicity in aposematic species, while accounting for the visual perception of relevant predators and evolutionary history where necessary, have uncovered contrasting results, even among closely related species (Darst et al. 2006; Cortesi and Cheney 2010; Wang 2011; Blount et al. 2012; Maan and Cummings 2012; Winters et al. 2014; Arenas et al. 2015; Crothers et al. 2016). Models attempting to reconcile these observations have focused on the economics of signal and defence, proposing that correlations or disjunctions in the costs of these two strategic components of aposematism will shape the relationship between them, with honesty arising when costs increase in parallel (Speed and Ruxton 2007). Nevertheless, a major obstacle to quantitative honesty in aposematism is the absence of a direct physiological link between signal and defence (Ruxton et al. 2004). The resource‐limitation model (Blount et al. 2009) potentially resolves this issue by suggesting that signals and defences may be competing for shared resources, whether energy in general or specific nutrients, such as carotenoids or other antioxidants. Going some way toward addressing these ideas, recent studies have begun to measure the physiological underpinnings of color signals and toxicity, such as hormone and carotenoid levels (Blount et al. 2012; Crothers et al. 2016) or sequestration ability (Mochida et al. 2013). However, more empirical work is needed to truly understand when and why honest signals may or may not be observed in nature.

In the case of signal honesty, relating theoretical models to empirical data is made more difficult by a mismatch in focus: while many modeling studies concentrate on variation within populations, relatively few studies have investigated this in the wild (Summers et al. 2015; Crothers et al. 2016). This study redresses the balance by focusing on variation within a single species, the six‐spot burnet moth (*Zygaena filipendulae* L.; Fig. 1A). *Zygaena* species are classic examples of aposematic Lepidoptera, combining striking red and black wing patterns with potent chemical defences, based on the cyanogenic glucosides linamarin and lotaustralin (Davis and Nahrstedt 1979, 1982; Fig. 1B). These compounds release hydrogen cyanide (HCN) when brought into contact with enzymes in larval haemolymph (Pentzold et al. 2017) or in the gut of predators. Found across the Western Palearctic (Naumann et al. 1999), *Z. filipendulae* is also locally abundant in Cornwall (southwest UK), enabling the collection of specimens from very distant populations in distinct habitat types, as well as of large samples from some local populations. Moreover, the cyanide‐based defences of *Z. filipendulae* have been extensively studied since they were first identified (Davis and Nahrstedt 1979), down to the genetic pathway controlling their synthesis (Zagrobelny et al. 2009; Jensen et al. 2011).

**Figure 1:**
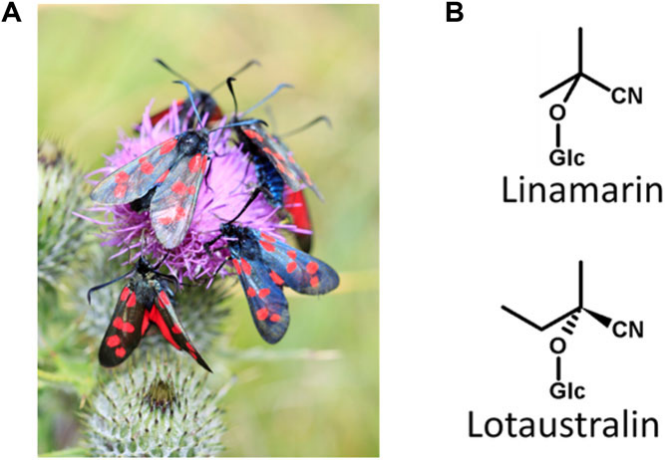
Six‐spot burnets, *Zygaena filipendulae* (A), and their cyanogenic glucoside defence compounds, linamarin, and lotaustralin (B).

Considering the importance of the relative costs of signals and defences and potential resource‐allocation trade‐offs for honest signaling to evolve, understanding the economics of defence in *Z. filipendulae* is crucial to predicting the relationship between color and toxicity in this species. Uniquely, among those insects tested to date, the larvae of *Zygaena* species can acquire the same defensive compounds by both sequestering them from their host plants and synthesising them *de novo* (Fürstenberg‐Hägg et al. 2014a). While larvae fed on acyanogenic host plants can largely compensate for the lack of these compounds in their diet, they develop more slowly, reach a lower mass at pupation, and incur a higher mortality than individuals fed on cyanogenic plants, confirming that *de novo* synthesis is energetically costly (Zagrobelny et al. 2007a). Nitrogen limitation is likely to be responsible for the trade‐off between investment in chemical defences and other functions: cyanogenic glucoside content decreases significantly during pupation, without release of HCN, suggesting that these compounds are broken down to fuel metamorphosis, and especially the synthesis of chitin, the main constituent of the cocoon and pupal case (Zagrobelny and Møller 2011; Fürstenberg‐Hägg et al. 2014b). In addition, the red colors of the Zygaeninae are generated by nitrogen‐rich pterin, or pteridine, pigments (Tremewan 2006), placing further pressure on nitrogen resources when producing signals and defences *de novo* (Morehouse and Rutowski 2010). Pterins are also known to have antioxidant functions, playing an important role in protecting immune cells (McGraw 2005), which raises the possibility of a trade‐off between antioxidant function, safeguarding against stored toxins, and pigmentation, as has been proposed for carotenoids (Blount et al. 2009, 2012).

This study examines the relationship between cyanogenic glucoside content and several measures of coloration to determine whether *Z. filipendulae* does display quantitatively honest signals, across 12 distinct localities, and in more detail within three populations. Under an honest signaling paradigm, higher levels of defensive compounds are expected to be associated with more conspicuous markings, likely in this case to be larger, redder, and more saturated (Stevens and Ruxton 2012). Variation in warning signals among and within populations were analysed independently, as the selective pressures contributing to diversity at these different levels are likely to vary (Summers et al. 2015). Within populations, predator learning may be important to the maintenance of signal honesty (Guilford and Dawkins 1993), while differences in the predator community, habitat, or other characteristics among populations could alter the relative costs of signals and defences (see Speed and Ruxton 2007). Along with precise quantification of toxins by LC‐MS, visual system‐dependent measures of coloration, based on models of avian vision, were used to assess variation in color as perceived by potential predators. As such, these results contribute relevant new insights into honest signaling within aposematic species.

## Methods

### SPECIMEN COLLECTION AND REARING

Ethical approval for the following experiments was granted by the University of Exeter (application reference number: 2015/1065). All individuals were collected at the larval or pupal stages, to ensure that freshly emerged, virgin moths were used, a critical consideration as males provide females with a nuptial gift of cyanogenic glucosides during mating, and females deposit these compounds in the eggs they lay (Zagrobelny et al. 2007a,b, 2013). Larvae and pupae of *Z. filipendulae* were collected in April to June 2015 and May 2016, from 12 sites in the United Kingdom, France, and Denmark (Supporting Information S1). The insects were reared in the laboratory until emergence of the adults, individually‐housed in plastic boxes with air holes, inside an incubator at 20°C with a 16 h day/night cycle, similarly to previous work on this species (Zagrobelny et al. 2007a). Larvae were reared with the same host‐plant species as they were found on in the field. For larvae from France, three different host‐plants were used (common bird's‐foot‐trefoil, *Lotus corniculatus* L., prostrate canary clover, *Dorycnium pentaphyllum* Scop., and horseshoe vetch *Hippocrepis comosa* L.); where possible cuttings from plants on local field sites were used, as well as *D. pentaphyllum* plants from a commercial nursery (Les Senteurs du Quercy, Mas de Fraysse, 46230 Escamps, France). Larvae found on *L. corniculatus* were fed cuttings of *L. corniculatus* from plants grown in greenhouses from commercially sourced plugs (Wildflower Shop, Elm House, Green Street, Suffolk, IP21 5AZ, UK). All larvae were fed *ad libitum*, with food replaced daily for freshness. A total of 107 adults emerged with undamaged wings and were used in subsequent photography and toxicity analyses (N_TOTAL_ = 107, N_DENMARK_ = 25, N_FRANCE_ = 18, N_UNITED KINGDOM_ = 64, with a range of 0–16 females and 0–19 males per collection site; see Supporting Information S1).

### WING PHOTOGRAPHY AND IMAGE ANALYSIS

As soon as the adults emerged and their wings were fully expanded, they were euthanized by placing them in a –80°C freezer. The sex and mass of each individual was determined, before their wings were dissected and photographed with a calibrated, UV‐sensitive digital camera (Nikon D7000 fitted with a 105 mm CoastalOptics quartz lens). Photographs were taken in controlled conditions in a dark room, illuminated by an EYE Color Arc Lamp MT70 bulb (Iwasaki Electric Co. Ltd), emitting a spectrum of light similar to D65 daylight conditions. Each image included a scale bar, label, and a set of reflectance standards, reflecting 7% and 93% of all wavelengths of light respectively (Zenith Lite Diffuse Target sheets, SphereOptics, Pro‐Lite Technology, Cranfield, UK), so as to further control for any variation in lighting conditions. As the wings of *Z. filipendulae* are iridescent, and thus the angle of incident light on the scales will affect the color of the wings, the light source was kept in a constant position, at a 50° angle relative to the wings, in all photographs. In addition, only the right‐hand wings were used for color measurements. Each specimen was photographed twice, using different filters: a UV/infrared blocking filter (Baader UV/IR Cut Filter), transmitting between 300 and 700 nm, and a UV pass and IR blocking filter (Baader U filter), transmitting between 300 and 400 nm (see Supporting Information S2). Combining these photographs yields a set of four image layers, corresponding to different parts of the visual spectrum: long wavelength (LW), medium wavelength (MW), short wavelength (SW), and ultraviolet (UV).

All subsequent image analysis was performed with a dedicated image calibration and analysis toolbox in ImageJ (Troscianko and Stevens 2015). To account for the camera's nonlinear response to different wavelengths of light, and changes in ambient light conditions (Stevens et al. 2007), images were linearized and normalised to the gray standards. The wing colors were then analysed from the perspective of potential predators, which in this case are most likely to be birds, with reports of burnet moth attacks attributed to a range of species, including blackbirds (*Turdus merula*), skylarks (*Alauda arvensis*), cuckoos (*Cuculus canorus*), house sparrows (*Passer domesticus*), starlings (*Sturnus vulgaris*), and meadow pipits (*Anthus pratensis*) (Tremewan 2006). Moth wing images were mapped to the two known broad categories of avian color visual system, which differ in the sensitivity of their most shortwave‐sensitive cone type, the ultraviolet‐sensitive (UVS), and violet‐sensitive (VS) groups (Hart et al. 1999), using data from their respective model species, the blue tit *Cyanistes caeruleus* (Hart et al. 2000) and peafowl *Pavo cristatus* (Hart 2002). With the same software package (Troscianko and Stevens 2015), linearized and normalized images were transformed to avian vision via a polynomial mapping technique with a D65 irradiance spectrum, which is highly accurate compared to cone catch modeling with reflectance spectra (Westland and Ripamonti 2004; Stevens and Cuthill 2006; Stevens et al. 2007; Pike 2011; Troscianko and Stevens 2015). This yielded five image layers, with predicted cone‐catch values for each photoreceptor type: ultraviolet‐ (UV‐ or V‐), short wavelength‐ (SW‐), medium wavelength‐ (MW‐), and long wavelength‐ (LW‐) sensitive photoreceptors, as well as the double cones. Wing markings and background areas on each photograph were selected using the freehand tool in ImageJ. While the position of the camera relative to each specimen was consistent for all photographs, all images were also scaled to 100 pixels/mm to eliminate any small discrepancies, which would affect size measurements. Each forewing spot was precisely outlined to allow for accurate measurements of its area, and if the spot was damaged, separate measurements of undamaged sections were taken for spot color. To measure the dark scales of the forewings and the red scales of the hindwings, zones as large as possible were selected, while avoiding damaged areas and creases. Cone catch values for every photoreceptor type were measured from each selected patch, then averaged to obtain a single measure of color per wing marking type. Analysis focused primarily on the moths’ red markings, as red coloration is a widespread and particularly effective aposematic signal (Stevens and Ruxton 2012; Arenas et al. 2014). However, the dark background colors were also measured, to calculate chromatic and luminance contrasts between the markings and background areas of each wing.

From the cone catch values, three metrics were calculated for the red markings of the fore‐ and hindwings: luminance, saturation, and hue. In brief, luminance provides a visual system‐dependent measure of perceived lightness (Osorio and Vorobyev 2005), while saturation and hue respectively describe the richness and shade of a color (Endler and Mielke 2005; Stevens et al. 2009). The perception of achromatic contrasts in birds is thought to be mediated by photoreceptors known as double cones, so luminance was taken as the cone catch value for the double cones (Jones and Osorio 2004; Osorio and Vorobyev 2005). To derive a measure of saturation, colors were plotted in a tetrahedral color space, with the coordinates corresponding to the proportion of total cone catch values to each channel: ultraviolet‐ (UV‐), short wavelength‐ (SW‐), medium wavelength‐ (MW‐), and long wavelength‐ (LW‐) sensitive (see Supporting Information S3 for the tetrahedral plot, produced in R with the plot.colspace function in the “pavo” package; Maia et al. 2013). Saturation corresponds to the Euclidean distance between the color of interest and the center of the color space (Endler and Mielke 2005; Stoddard and Prum 2008). Finally, as in a range of previous studies of animal coloration (Komdeur et al. 2005; Spottiswoode and Stevens 2011; Stevens et al. 2014), estimates of hue were broadly based on the concept of opposing color channels. Similarly to human vision, opponent mechanisms are likely to be important for processing color signals in birds (Osorio et al. 1999), although the exact opponent channels are not precisely known. Nevertheless, principal component analysis (PCA) can be used to estimate the principal axes of variation in color between samples. Following Spottiswoode and Stevens (2011), we performed PCA on a covariance matrix of the standardized values of all color patches for the four photoreceptor channels (UV, SW, MW, LW) for each visual system. The first principal component obtained accounted for 81–95% of the variance in marking color. It was used to calculate a ratio of cone catch values, forming a logical color channel, which was identical for both fore‐ and hindwing markings, and both visual systems:

(1) $$\mathrm{Hue}\;=\frac{LW}{\left(UV+SW+MW\right)/3}$$

UV,SW,MW,LW= standardized cone catch values for the UV‐, SW‐, MW‐, and LW‐ sensitive photoreceptors, respectively.

Although this channel does not represent an actual opponent channel, this ratio provides a meaningful and intuitive measure of hue, broadly inspired by opponent mechanisms. High values of hue correspond to colors with relatively greater reflectance in the long wavelength (LW) color channel than in the short, medium, and ultraviolet wavelength channels (SW, MW, UV), so represent redder colors.

In addition to the metrics above, two measures of visual contrast were calculated to provide data on the perceived differences between red and black areas on the moths’ forewings and hindwings. The salience of these internal contrasts can constitute an important feature of warning signals, affecting predator learning (Aronsson and Gamberale‐Stille 2012; Barnett et al. 2016). Chromatic contrast was calculated according to a widely used log version of the receptor noise‐limited Vorobyev‐Osorio color discrimination model (Vorobyev and Osorio 1998), which takes into account the sensitivity and abundance of each cone type (relative cone abundance taken as UV = 1, SW = 1.92 MW = 2.68, LW = 2.7 for the UVS system (Hart et al. 2000) and V = 1, SW = 1.9, MW = 2.2, LW = 2.1 for the VS system (Hart 2002; Håstad et al. 2005)), as well as the noise in the photoreceptors. Noise was calculated with a relatively conservative estimate of the Weber fraction, ω=0.05, for the most abundant cone type (Eaton 2005; Håstad et al. 2005; Stevens 2011; Stevens et al. 2014). Luminance contrast was computed as the natural logarithm of the ratio between mean double cone catch values of background and marking areas, divided by the same Weber fraction (Siddiqi et al. 2004). Contrast values are measured in “just‐noticeable differences,” or JNDs: values less than 1 mean that two colors should be indiscriminable, and increasing values indicate that colors are likely to be increasingly easy to distinguish (Siddiqi et al. 2004).

### CONTRAST TO NATURAL BACKGROUNDS

Conspicuousness of prey against the natural backgrounds on which they are found is a key component of aposematic signaling (Stevens and Ruxton 2012; Arenas et al. 2015; Arenas and Stevens 2017), which should more often be measured in empirical studies of honest signaling (Arenas et al. 2015). To address this issue, chromatic and luminance contrasts were calculated between the moth's wing markings and three likely natural backgrounds: the leaves and flowers of their principal host plant, *Lotus corniculatus* (Fabaceae), and a popular nectaring flower, field scabious (*Knautia arvensis*, Dipsacaceae) (Naumann et al. 1999; Zagrobelny et al. 2015). Five plants of each species were collected for analysis in Cornwall (UK). One leaf or flower was taken from each of these plants and photographed with the same equipment and under the same conditions as the moth wings (total of five samples per plant type). *L. corniculatus* flowers were dissected so that the upper and lower petals could be photographed as flat as possible. Plant areas for analysis were once again selected using the freehand tool in Image J: each of the three leaflets of every *L. corniculatus* leaf, each petal from the *L. corniculatus* flowers, and three outer petals and the central area of *K. arvensis* flowers, for every sample. Color measurements were then averaged to obtain a single value per plant species and tissue type (flower or leaf), and contrasts between these values and those of the moth forewing markings were calculated using chromatic and luminance JNDs, as described above.

### QUANTIFICATION OF CYANOGENIC GLUCOSIDES

Measures of cyanogenic glucoside levels were obtained with a liquid chromatography‐mass spectrometry (LC‐MS) technique refined for detecting cyanogenic glucosides such as linamarin and lotaustralin in extracts from plants and insects, and previously employed in numerous studies of chemical defences in *Z. filipendulae* (Zagrobelny et al. 2004, 2007a,b, 2014, 2015; Fürstenberg‐Hägg et al. 2014b; Pentzold et al. 2015, 2016). Prior to LC‐MS analysis, the frozen samples were each ground up in 1ml ice‐cold 55% MeOH, containing 0.1% formic acid and 0.044 mM amygdalin, a cyanogenic glucoside not found in the Zygaenidae, as an internal standard. All samples were subsequently passed through an Anopore 0.45 μm filter (Whatman) and analytical LC‐MS was carried out using an Agilent 1100 Series LC (Agilent Technologies, Germany), interfaced with a Bruker HCT‐Ultra ion trap mass spectrometer (Bruker Daltonics, Bremen, Germany). Chromatographic separation was performed with a Zorbax SB‐C18 column (Agilent; 1.8 μM, 2.1 × 50 mm) at a flow rate of 0.2 ml/min, increased to 0.3 ml/min from 11.2 to 13.5 min. Oven temperature was maintained at 35°C and the mass spectrometer was run in positive electrospray mode. The mobile phases were A (H_2_O with 0.1% v/v HCOOH, 50 μM NaCl) and B (MeCN with 0.1% v/v HCOOH), with a gradient program as follows: 0–0.5 min, isocratic 2% B; 0.5 to 7.5 min, linear gradient 2–40% B; 7.5–8.5 min, linear gradient 40–90% B; 8.5–11.5 isocratic 90% B; 11.6–17 min, isocratic 2% B. Mass spectral data were analyzed with the native data analysis software, to detect sodium adducts of linamarin (retention time (RT) 2.6 min, [M+Na]^+^ at *m/z* 270), lotaustralin (RT 5.5 min, [M+Na]^+^ at *m/z* 284), and amygdalin (RT 6.6 min, [M+Na]^+^ at *m/z* 480) and compare them to authentic standards (Møller et al. 2016). The total amount of each compound was estimated according to its extracted ion chromatogram (EIC) peak areas and quantified based on calibration curves of linamarin, lotaustralin, and amygdalin standards.

### STATISTICAL ANALYSES

All results were analysed using R 3.3.1 (R Development Core Team 2015). Forewing and hindwing data were treated separately, and all analyses were repeated with data from both the UVS (blue tit) and VS (peafowl) visual systems. Among populations, the relationship between mean cyanogenic glucoside levels and mean color values between populations was examined, following a similar approach to a previous study of signal honesty among poison frog populations (Maan and Cummings 2012). Linear models testing the relationship between toxin levels and each color metric were run using the lm function in R, and model assumptions were verified with diagnostic plots. These analyses were run for each sex separately, as varying numbers of males and females were sampled in each population, potentially affecting the outcome of models based on a single average per population.

To explore the question of honesty in aposematic signaling within populations, data from three populations (Holywell Bay, UK, Lamorna Cove, UK, and Taastrup, D., where *N* > 20) were investigated in more detail. Multiple linear regression was used to test the relationship between the concentration of cyanogenic glucosides in each sample and wing coloration, in each population separately. Each model included all relevant color metrics for either the forewing or hindwing markings, with one exception. Saturation and hue values were calculated from the same cone catch values, so as expected, were highly correlated (Pearson's correlation > 0.99, calculated with the cor.test function). Linear regression models thus included either saturation or hue, to avoid the problem of high collinearity in the analysis; models including only one of these measures of color yielded the same conclusions (Supporting information S4) and had variance inflation factors (VIFs, calculated using the vif function in the “car” package in R (Fox and Weisberg 2011)) below the recommended threshold of 10 (Dormann et al. 2013). Stepwise model simplification was carried out to identify the minimal model in each case, based on likelihood tests carried out with the ANOVA function, and a significance threshold of α = 0.05. If any outliers were identified (Cook's distance >1 in diagnostic plots), the models were run with and without these datapoints to test their influence; results are only reported without outliers if their removal significantly affected the model output. Two outliers with cyanogenic glucoside levels 10‐fold lower than the mean were removed from the Lamorna cove dataset, as they substantially influenced results. The assumptions of all linear models were verified using diagnostic plots.

Sex differences in coloration in these three populations were also investigated using linear models and the lm function, with each color metric a dependent variable and allowing population and sex to interact as independent variables. Tukey's post‐hoc tests were implemented to determine significant pairwise comparisons between populations, using the glht function in the “multcomp” package in R (Hothorn et al. 2008). Finally, contrasts between forewing markings and natural backgrounds were analyzed with linear‐mixed models, with sex, population, and plant type as fixed effects and individual ID as a random effect, using the lmer function in the package “lme4” (Bates et al. 2014). Model diagnostics were checked using the mcp.fnc function in the “LMERConvenienceFunctions” package (Tremblay and Ransijn 2014). To fit model assumptions, luminance contrast was transformed with the logit function in the “car” package (Fox and Weisberg 2011). Pairwise comparisons between populations and plant background type were again tested with Tukey's post‐hoc tests using the glht function (Hothorn et al. 2008).

## Results

For clarity, the results reported below are based on the ultraviolet‐sensitive (UVS, or blue tit) visual system only. Despite some discrepancies, particularly greater differences in coloration between two populations (Lamorna Cove and Taastrup), results for the violet‐sensitive (VS, or peafowl) visual system are generally qualitatively similar and support the findings based on the UVS model; details can be found in Supporting information S5 and S6.

### VARIATION AMONG POPULATIONS

**Figure 2:**
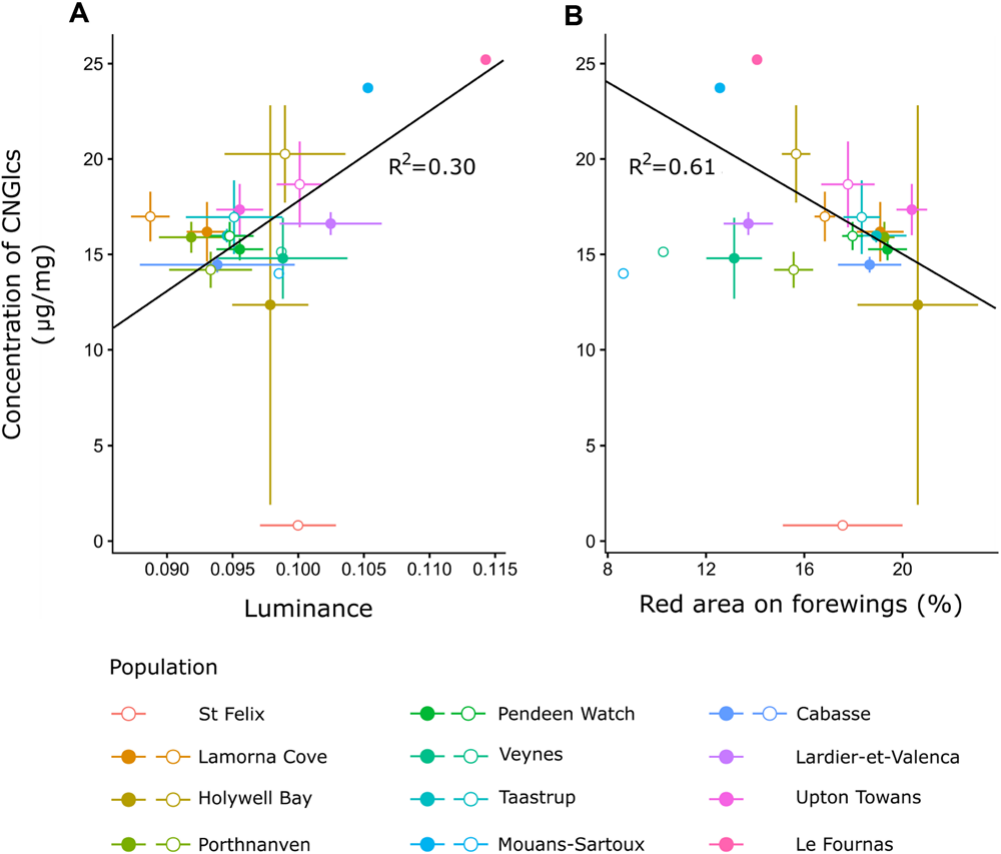
Mean cyanogenic glucoside concentration and luminance (A) and relative spot size (B) across populations, for males (open circles) and females (full circles). Error bars correspond to standard errors for both color metrics and toxin concentration. CNGlcs = cyanogenic glucosides. Lines represent the linear relationship between color metrics and cyanogenic glucoside concentration for females.

There was no significant relationship between color metrics and toxin concentration among populations for males (Table 1). In addition, measures of hindwing coloration were not significantly associated with cyanogenic glucoside levels in either sex. However, for females, an increase in mean concentration of these compounds per population was associated with higher luminance and smaller relative spot area (Fig. 2, Table 1), suggesting that higher toxin levels were represented by lighter and smaller markings.

**Table 1:** Relationship between color metrics and cyanogenic glucoside concentrations across populations

Color metric	Males	Females
FW luminance	*F*_1,7_ = 0.45, *P* = 0.52, *R* ^2^ = –0.074	*F*_1,9_ = 16.47, *P* = 0.0029, *R* ^2^ = 0.61
FW saturation	*F*_1,7_ = 0.24, *P* = 0.64, *R* ^2^ = –0.11	*F*_1,9_ = 4.35, *P* = 0.067, *R* ^2^ = 0.25
FW hue	*F*_1,7_ = 0.11, *P* = 0.75, *R* ^2^ = –0.12	*F*_1,9_ = 4.06, *P* = 0.075, *R* ^2^ = 0.23
FW chromatic contrast	*F*_1,7_ = 0.81, *P* = 0.397, *R* ^2^ = –0.0240	*F*_1,9_ = 5.03, *P* = 0.052, *R* ^2^ = 0.29
FW luminance contrast	*F*_1,7_ = 0.41, *P* = 0.54, *R* ^2^ = –0.079	*F*_1,8_ = 1.06, *P* = 0.33, *R* ^2^ = 0.0064
Proportion of red in FWs	*F*_1,7_ = 0.003, *P* = 0.96, *R* ^2^ = –0.14	*F*_1,9_ = 5.25, *P* = 0.048, *R* ^2^ = 0.30
HW luminance	*F*_1,7_ = 0.29, *P* = 0.61, *R* ^2^ = –0.097	*F*_1,9_ = 1.78, *P* = 0.22, *R* ^2^ = 0.073
HW saturation	*F*_1,7_ = 0.18, *P* = 0.68, *R* ^2^ = –0.11	*F*_1,9_ = 0.0005, *P* = 0.98, *R* ^2^ = –0.11
HW hue	*F*_1,7_ = 0.091, *P* = 0.77, *R* ^2^ = –0.13	*F*_1,9_ = 0.0047, *P* = 0.95, *R* ^2^ = –0.11

Significant results are in italics. A relatively high *R* ^2^ value for the relationship between toxicity and luminance in females suggests this may be the most relevant result, while low *R* ^2^ values for relationships with *P*‐values near the significance threshold (*P* < 0.05) indicate that these are less likely to be biologically important. FW = forewing, HW = hindwing.

### VARIATION WITHIN POPULATIONS

#### Coloration and cyanogenic glucoside concentration

**Figure 3:**
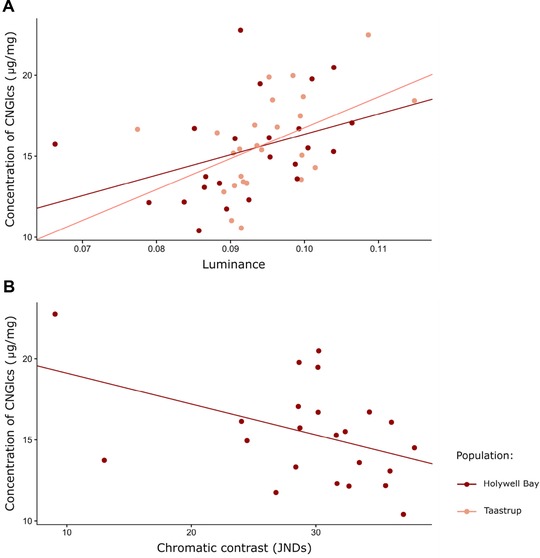
Relationship between forewing luminance (A), chromatic contrast (B), and the concentration of cyanogenic glucosides, in the Holywell Bay and Taastrup populations. CNGlcs = cyanogenic glucosides.

In both the Holywell Bay and Taastrup populations, forewing luminance was positively associated with cyanogenic glucoside concentration (linear models, luminance: F_1,20_ = 4.36, *P* = 0.050, and F_1,23_ = 6.77, *P* = 0.016, respectively; Fig. 3A, Supporting information S4). In the Holywell Bay sample, cyanogenic glucoside concentration was also negatively associated with chromatic contrast between the forewing background and marking colors (linear model, chromatic contrast: F_1,20_ = 5.64, *P* = 0.028; Fig. 3B). In contrast, in the Lamorna Cove dataset, there were no significant relationships between glucoside levels and any color metrics (Supporting information S4).

#### Sexual dimorphism

**Figure 4:**
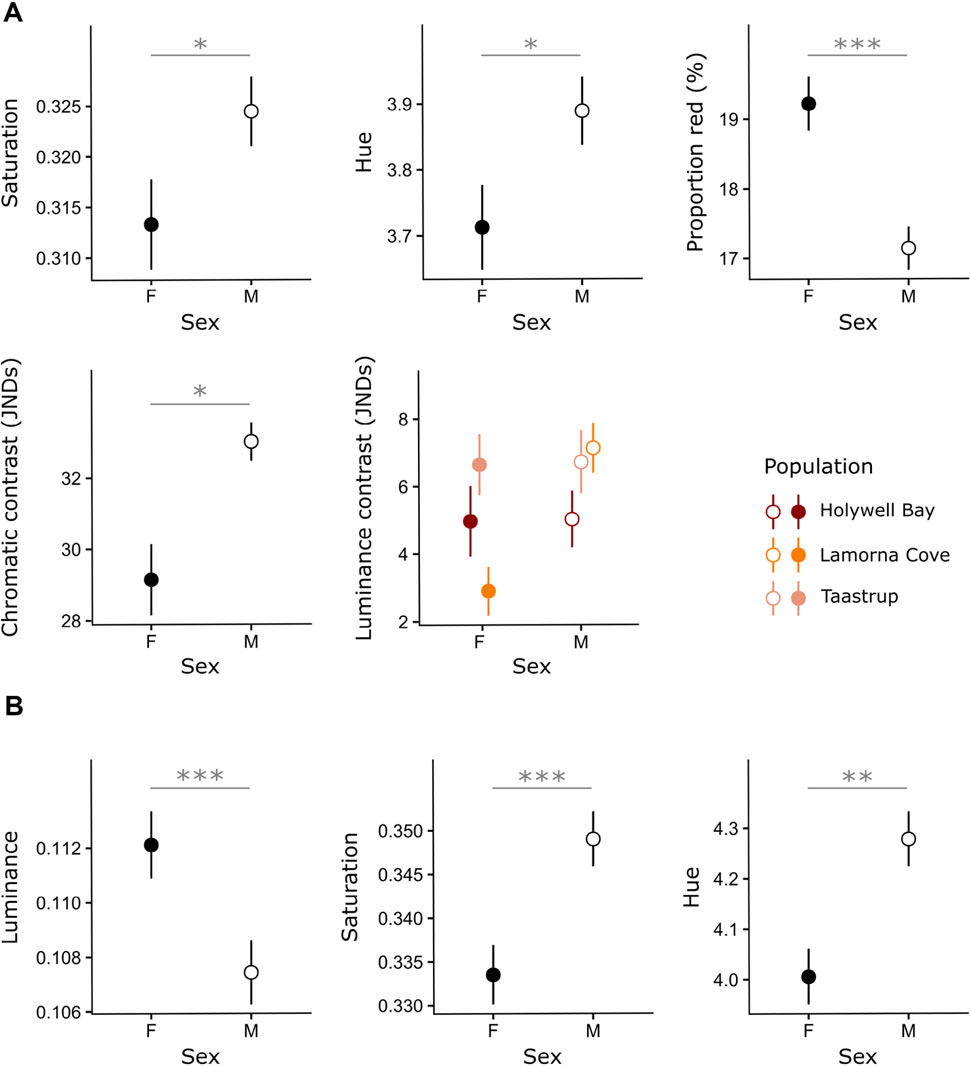
Mean and standard error for color metrics in the forewings (A) and hindwings (B) of specimens from Holywell Bay, Lamorna Cove, and Taastrup. Closed circles represent females, open circles males. Luminance contrast is plotted by population, as the relationship between sex and this metric varied between localities. Significance levels: ^***^ *P* < 0.001, ^**^ *P* < 0.01, ^*^ *P* < 0.05.

There was no difference in the concentration of toxins between sexes or populations (linear model, sex: F_1,69_ = 0.0002, *P* = 0.99; population: F_2,70_ = 0.70, *P* = 0.50). However, the total amount of cyanogenic glucosides did differ, due to size dimorphism: larger females possessed consistently greater amounts of these compounds than males, in all three populations (linear model, sex: F_1,69_ = 107.31, *P* < 0.001). Moreover, males and females differed in all color metrics, with the exception of forewing luminance. Saturation, hue, and chromatic contrast of the red forewing markings were higher in males than females, while female markings were larger relative to total wing area (Table 2A, Fig. 4A). Forewing luminance contrast was higher in males than females in Lamorna Cove, but not in the other localities (Table 2A, Fig. 4A). In the hindwings, luminance was higher in females, but saturation and hue values were again greater in males (Table 2B, Fig. 4B). Populations also differed overall in some metrics: chromatic contrast was higher in Lamorna Cove than in the Taastrup population (Tukey's HSD: p_Lamorna‐Holywell_ = 0.12, p_Taastrup‐Holywell_ = 0.90, p_Lamorna‐Taastrup_ = 0.033) and hindwing luminance was lower in the Lamorna Cove population than in the others (Tukey's HSD: p_Lamorna‐Holywell_ = 0.0094, p_Taastrup‐Holywell_ = 1, p_Lamorna‐Taastrup_ = 0.0074).

**Table 2:** Results of linear models examining sex and population differences in color metrics

a. In the forewings									
Factor	F	df	P	F	df	P	F	df	P
	Luminance			Saturation			Hue		
Sex:population	0.84	2.67	0.44	1.17	2.67	0.32	1.28	2.67	0.28
Population	2.39	2.69	0.099	1.94	2.69	0.15	1.35	2.69	0.27
Sex	0.082	1.71	0.77	4.073	1.71	0.047	4.70	1.71	0.034

	Proportion red			Chromatic contrast			Luminance contrast		
Sex:population	1.51	2.67	0.23	0.47	2.67	0.62	4.57	2.67	0.014
Population	2.28	2.69	0.11	3.79	2.69	0.028	—	—	—
Sex	17.77	1.71	<0.001	12.90	1.69	<0.001	—	—	—

b. In the hindwings
Factor	F	df	P	F	df	P	F	df	P
	Luminance			Saturation			Hue		
Sex:population	0.25	2.67	0.77	1.21	2.67	0.31	1.18	2.67	0.31
Population	6.61	2.69	<0.01	1.01	2.69	0.37	0.69	2.69	0.51
Sex	16.20	1.69	<0.001	36.19	1.71	<0.001	12.19	1.71	0.001

Significant results are highlighted in italics.

#### Conspicuousness against natural backgrounds

**Figure 5:**
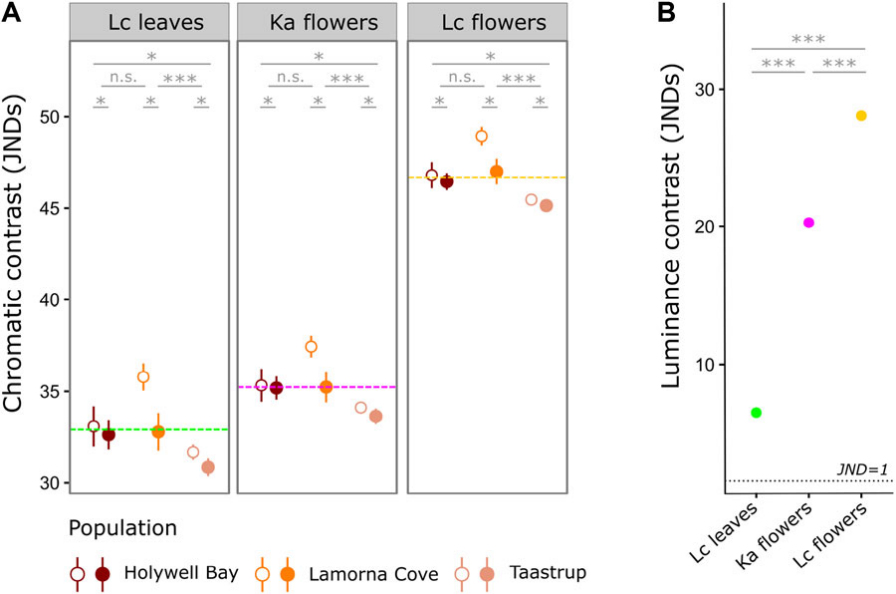
Mean and standard errors for chromatic (A) and luminance (B) contrast between forewing markings and natural backgrounds. In (A), closed circles represent females, open circles males, and dashed lines represent the mean chromatic contrast for each plant type. Lc = *Lotus corniculatus*, Ka = *Knautia arvensis*. Significance levels: ^***^ *P* < 0.001, ^**^ *P* < 0.01, ^*^ *P* < 0.05.

Chromatic contrast to plant tissues on which adult *Z. filipendulae* are likely to be observed was higher in males than females (LME, sex: (χ^2^)_1_ = 4.75, *P* = 0.029), and lowest overall in the Taastrup population (LME, population: (χ^2^)_2_ = 20.23, *P* = 0.000040, Tukey's HSD: p_Lamorna‐Holywell_ = 0.16, p_Taastrup‐Holywell_ = 0.03, p_Taastrup‐Lamorna_ < 0.0001; Fig. 5A). However, luminance contrast did not vary according to population or sex (LME, sex × population: (χ^2^)_2_ = 1.67, *P* = 0.43; sex: (χ^2^)_1_ = 0.48, *P* = 0.49; population: (χ^2^)_2_ = 4.43, *P* = 0.11). The forewing markings were consistently least conspicuous against the leaves of their hostplant, *Lotus corniculatus*, and most conspicuous against its flowers (LME, plant type: (χ^2^)_2_ = 768.12, *P* < 2.2 × 10^−16^ and (χ^2^)_2_ = 705.96, *P* < 2.2 × 10^−16^ for chromatic and luminance contrast, respectively; Tukey's HSD, *P* < 2.2 × 10^−16^ for all pairwise comparisons; Fig. 5). Nevertheless, contrast values were consistently greater than the threshold for discrimination (JND = 1), and were especially high in chromatic terms, making all forewing markings conspicuous, regardless of population, sex, and plant type differences.

## Discussion

The principal aim of this study was to test for quantitative honesty in the warning signals of *Z. filipendulae*. It is important to note that all individuals were highly toxic and conspicuous, so any differences between individuals, sexes, and populations could merely act to provide more detailed information about the level of defence. With few exceptions, trends were broadly similar for both types of avian visual system, so the conclusions drawn here are likely applicable to a variety of potential avian predators of *Z. filipendulae*. Across populations, significant relationships between measures of color and toxin levels were only found in female specimens, for whom higher concentrations of cyanogenic glucosides were associated with lighter and smaller markings. Within populations, sex appeared to be the primary determinant of coloration: female markings were larger, but also lighter, less saturated, less red and less contrasting than those of males. Few color metrics were significantly associated with cyanogenic glucoside concentration within populations: positive relationships between toxin levels and luminance were found in two localities, as well as, for the UVS visual system alone, a negative relationship between toxicity and chromatic contrast in a single population. Collectively, these results primarily indicate a lack of quantitative honesty linking measures of color and toxin concentration, at both the individual and population levels. Individuals with greater cyanogenic glucoside levels did not display features typically considered to increase signal salience, instead appearing paler, less saturated, and less red than less well‐defended specimens.

Disentangling the specific roles of chromatic and achromatic information in influencing predator behavior, along with specific colors, internal contrasts and conspicuousness against natural backgrounds, is an important area for future research in the field of visual communication (Stevens and Ruxton 2012). In this study, while most color metrics are quantitatively unrelated or negatively related to defence levels, larger females do display larger markings than males, which could be a useful cue for predators, and the lighter markings of individuals possessing higher levels of cyanogenic glucosides could be interpreted as an honest signal. Among populations, relationships between defence levels and internal contrasts in the forewings further support a positive association of increased defences with achromatic features, and a negative association with chromatic measures of signal strength, but these results only appear significant for females, and when considering the VS visual system. Moreover, all specimens were highly conspicuous, so it is unclear whether avian predators would discriminate between individuals with small, albeit perceptible, differences in coloration. While taking these limitations into account, in the absence of experimental evidence of predator responses to variation in the different components of zygaenid warning signals, the results presented here overall do not support the idea of quantitative honesty in this species.

### COLORATION, DEFENCE, AND RESOURCE ALLOCATION TRADE‐OFFS

Several empirical studies in other taxa (e.g., in Pachycephalidae songbirds [Dumbacher et al. 2000, 2008] and the Japanese fire‐bellied newt, *Cynops pyrrhogaster* [Mochida et al. 2013]) have previously found either no relationship or negative correlations between aposematic signals and the strength of the defences they are advertising, including both within and between populations of single species of poison frogs (Daly and Myers 1967; Wang 2011; Crothers et al. 2016). Populations of orange and green *Oophaga granulifera* (Dendrobatidae), less contrasting to their natural backgrounds according to avian vision, were found to possess greater levels of toxic alkaloids than more conspicuous red populations (Wang 2011). In this case, migration of populations into areas where more potent alkaloids were available in the poison frog diet is thought to have driven a subsequent reduction in visual conspicuousness, reflecting a strategic trade‐off between signals and defences in aposematism (Darst et al. 2006; Speed and Ruxton 2007). The aversiveness of highly toxic prey will in itself stimulate predator learning, reducing the incentive for displaying obvious signals, which also carry costs, such as visibility to naïve predators. According to the predictions of the resource‐limitation model of signal honesty in aposematism, if resources are plentiful, so toxins can be acquired cheaply, prey should invest primarily in these rather than visual signals, while signals should be honest when resources are limited (Blount et al. 2009).

Strategic trade‐offs may also explain the negative relationships between multiple measures of coloration and pumiliotoxin levels within the highly toxic and conspicuous Solarte population of *Oophaga pumilio*. The strong selective pressure to avoid these well‐defended frogs may encourage predator generalization, such that predators may broadly avoid prey that resembles the Solarte morph, even if there is variation in their warning signals. The frogs could thus maintain effective visual deterrents to predation while reducing their investment in coloration (Crothers et al. 2016). This situation draws parallels with *Z. filipendulae*, which are also very conspicuous and especially toxic among Lepidoptera (Rothschild et al. 1970; Sbordoni et al. 1979). For such unprofitable prey, there may be little to gain by communicating additional information to predators by the means of quantitatively honest signals, since anything resembling the toxic prey will be strongly avoided. Variation in the strength of defences among aposematic species, and hence in the risks incurred by predators feeding on these types of prey, could be an important factor explaining the contrasting evidence for quantitative signal honesty uncovered by empirical studies so far. While experienced predators may always try to avoid all individuals of very highly defended species, honest signaling in species with relatively weak defences may be genuinely informative to predators, such that a stronger signal may confer greater protection on a well‐defended individual. As a result, we may be more likely to find evidence of quantitative honesty in less strongly defended species. However, this does not account for the possibility that in some foraging instances a predator may consume only a proportion of the prey, meaning that even highly toxic species may be nonlethal. Greater understanding of the unprofitability of different aposematic species to avian predators and foraging behavior would be helpful in testing this hypothesis. Alternatively, differences in predator communities between populations (Endler and Mappes 2004), or effective generalization due to perceptual limitations of the predators could contribute to the trends found in poison frogs, and here in *Z. filipendulae*.

### SEXUAL DIMORPHISM AND SEXUAL SIGNALING

More clearly than cyanogenic glucoside levels, sex emerged as a key factor underlying variation in appearance in *Z. filipendulae*, in all studied populations. Differences in activity patterns between sexes may expose them to unequal predation pressures, as more active males, flying to seek out females (Naumann et al. 1999), may be more likely to encounter predators and hence benefit from investing more in conspicuous warning signals. However, *Z. filipendulae* often occur in large numbers and calling females are also highly conspicuous, exposed on flowers such as *K. arvensis* (Zagrobelny et al. 2013; pers obs.). Perhaps more relevant is the size dimorphism between males and females: for males, redder and more saturated markings might compensate for their smaller marking size, improving their salience to predators. By contrast, females may benefit from prioritising investment in toxins to ensure protection, as predators balance the risk of consuming toxic prey with the nutritional benefit gained from consuming larger, more nutritious prey (Smith et al. 2016).

Evidence of sexual dichromatism also raises the possibility that color could be involved in sexual selection and mate choice. While pheromones are recognized as the principal means for intraspecific communication in the Zygaenidae, several observational and experimental studies suggest that visual cues might also be relevant in certain species, including *Z. filipendulae* (reviewed in Subchev 2014; Sarto i Monteys et al. 2016). Both *Z. filipendulae* and *Z. trifolii*, potentially along with other European species (Hofmann and Kia‐Hofmann 2010), are thought to employ two alternative mating strategies, with males relying on pheromone plumes to locate calling females in the afternoon, but using visual cues to find mates in the morning, when females are not producing pheromones (Naumann et al. 1999; Subchev 2014). Multiple cues might also be used in different phases of mate localization: males of *Z. filipendulae* are thought to rely on pheromone plumes to locate mates, then use visual cues to orient themselves at close range, approximately 50 cm away from the females (Zagatti and Renou 1984), a strategy also seen in *Z. niphona* and *Z. fausta* (Koshio 2003; Friedrich and Friedrich‐Polo 2005; Sarto i Monteys et al. 2016). Studies seeking to manipulate visual cues during courtship in the Zygaenidae have found some limited evidence for their use in male mate choice (Zagatti and Renou 1984; Toshova et al. 2007). Monitoring the approach and copulatory attempts of wild *Z. filipendulae* males to an array of artificial female stimuli revealed that, although males did not discriminate between mounted female specimens of closely related species, the presence of red coloration generally encouraged copulation, and fresher specimens were preferred, leading the authors of this study to conclude that males favor more saturated colors (Zagatti and Renou 1984). Although the males were likely to perceive the crude differences between the artificial baits used in that study, little is known about visual perception in the Zygaenidae, so more systematic discrimination tests would be needed to establish whether color difference on the scale measured here could be relevant to mate choice. Moreover, we found no evidence of positive relationships between color and cyanogenic glucoside levels in either sex in natural populations, so color would not provide quantitative information about the defences of potential mates. However, male preference for more saturated colors could be relevant to the absence of quantitative honesty between color and the levels of defensive compounds at emergence in this species.

### VARIABLE AND MULTIMODAL SIGNALING

Over the lifetime of an adult burnet moth (and many Lepidoptera in general), wing scales are progressively brushed off, such that older individuals are visibly faded (pers. obs.). In the orange sulphur butterfly (*Colias eurytheme*), wing colors fade with age (Kemp 2006), and the decline in UV reflectance in particular may help females select younger males (Papke et al. 2007), an advantageous strategy as male age is negatively correlated with the protein content of nuptial gifts in this and other butterfly species (Rutowski and Gilchrist 1986; Rutowski et al. 1987). In *Z. filipendulae*, females receive nuptial gifts of cyanogenic glucosides (Zagrobelny et al. 2007a) and are known to reject smaller and less well‐defended suitors (Zagrobelny et al. 2007a, 2013), a bias which can be overcome if the males are injected with extra cyanogenic glucosides or painted with linamarin (Zagrobelny et al. 2015). While females are thought to use compounds deposited on male abdominal brushes, or corremata, to assess male quality, chemical cues are not always reliable: in fact, males emit higher levels of HCN after mating, due to the presence of residual compounds on their corremata, despite having fewer cyanogenic glucosides left to offer (Zagrobelny et al. 2007a, 2015). Since the cyanogenic glucoside reserves of older males are more likely to have been depleted by successive matings, wing color could assist female choice as a useful proxy for male age. Likewise, female *Z. filipendulae* can mate multiple times (Naumann et al. 1999), but males will benefit from mating with younger females, with a greater number of eggs available for fertilization. As a result, both sexes should prefer younger mates, and brighter, more saturated wing colors could act as reliable indicators of quality. From a predator's perspective, wing color could similarly be used as a crude signal of toxin content in the wild. Taking toxin and color measurements from a range of individuals at a given date in any given population, effectively a snapshot of prey items available to predators, would help test this hypothesis.

Rather than focusing on visual signals alone, observations in *Z. filipendulae* demonstrate the importance of considering these as elements of a more complex multimodal and multicomponent signaling system (see Rowe and Halpin 2013). In this study, we showed that the red markings of *Z. filipendulae* do not function as straightforward quantitatively honest signals of the levels of defensive compounds, neither within nor between populations. The very high defence levels of this species, potentially reducing the usefulness of quantitative honesty in signaling, and the fragile nature of wing color on the moths’ wings, fading rapidly with time, are important considerations potentially explaining these results. However, color signals are only one means of communication employed by the Zygaenidae; visual cues are likely to be used in combination with pheromone emission, deposits on corremata and, for predators, with the bitter taste of the cyanogenic glucosides, to evaluate the profitability of individuals. Further research into the volatiles emitted by zygaenids, including degradation products of cyanogenic glucosides such as HCN and ketones (Zagrobelny et al. 2015) and pyrazines (Rothschild 1961; Rothschild et al. 1984; Moore et al. 1990; Tremewan 2006), odors often associated with warningly colored insects (Guilford et al. 1987), will help develop a more comprehensive picture of their defensive strategy. Testing the response of natural predators to these volatiles, as well as taste‐rejection due to the bitter cyanogenic glucosides, and how these cues interact with each other and visual signals, are the next logical steps to establishing the relevance of these strategic components to survival in the wild. In this and other study systems, integrating the effects of multiple cues, especially visual and chemical, is a major route toward a deeper understanding of aposematic signaling strategies.

Associate Editor: G. Höbel

Handling Editor: P. Tiffin

## Supporting information

Table S1: Coordinates of sites on which *Z. filipendulae* larvae and pupae were collected.
Table S5: Relationship between color metrics and cyanogenic glucoside concentrations across populations.
Table S6a: Results of multiple regressions exploring the relationship between cyanogenic glucoside concentration and color metrics in the forewings (i) and hindwings (ii).
Table S6b: Results of linear models testing for sex and population‐level differences in color metrics across Holywell Bay, Lamorna Cove and Taastrup.
Table S6c: Results of mixed models testing differences in contrast between forewing markings in Holywell Bay, Lamorna Cove and Taastrup, and natural backgrounds.
Figure S1b: Map of collection sites; numbers represent specimens photographed.
Figure S2: Photographs of one individual's wings, taken with the UV/infrared blocking filter (a) and the UV pass and IR blocking filter (b).
Figure S3: Plots of all individuals’ red area colors in the forewing (a) and hindwing (b) in a tetrahedral color space, for the UVS visual system.
