# Supplementary material for: Sex differences but no evidence of quantitative honesty in the warning signals of six‐spot burnet moths (Zygaena filipendulae L.)
Source: Evolution. 2018 Jul 2;72(7):1460–74. doi: 10.1111/evo.13505 (PMC6099377; doi:10.1111/evo.13505)
Supplement: Supplementary file 1 — Table S1: Coordinates of sites on which Z. filipendulae larvae and pupae were collected. Table S5: Relationship between color metrics and cyanogenic glucoside concentrations across populations. Table S6a: Results of multiple regressions exploring the relationship between cyanogenic glucoside concentration and color metrics in the forewings (i) and hindwings (ii). Table S6b: Results of linear models testing for sex and population‐level differences in color metrics across Holywell Bay, Lamorna Cove and Taastrup. Table S6c: Results of mixed models testing differences in contrast between forewing markings in Holywell Bay, Lamorna Cove and Taastrup, and natural backgrounds. Figure S1b: Map of collection sites; numbers represent specimens photographed. Figure S2: Photographs of one individual's wings, taken with the UV/infrared blocking filter (a) and the UV pass and IR blocking filter (b). Figure S3: Plots of all individuals’ red area colors in the forewing (a) and hindwing (b) in a tetrahedral color space, for the UVS visual system. [file EVO-72-1460-s001.docx]

**SUPPORTING INFORMATION**

**S1: Collection sites**

Table S1: Coordinates of sites on which *Z. filipendulae* larvae and pupae were collected.

| Site name | Country | Latitude | Longitude | Altitude (m) | N moths | | |
| --- | --- | --- | --- | --- | --- | --- | --- |
|  |  |  |  |  | **Female** | **Male** | **TOTAL** |
| Taastrup | Denmark | 55.6346 | 12.2625 | 30 | 6 | 19 | 25 |
| Cabasse | France | 43.4201 | 6.2355 | 250 | 2 | 2 | 4 |
| Lardier-et-Valenca | France | 44.2512 | 5.5689 | 830 | 5 | 0 | 5 |
| Le Fournas | France | 44.0753 | 5.9722 | 480 | 1 | 0 | 1 |
| Mouans-Sartoux | France | 43.6204 | 6.9725 | 150 | 1 | 1 | 2 |
| St Félix de Tournegat | France | 43.1312 | 1.7483 | 310 | 0 | 2 | 2 |
| Veynes | France | 44.3239 | 5.4924 | 900 | 3 | 1 | 4 |
| Holywell Bay | United Kingdom | 50.3910 | -5.1430 | 20 | 16 | 7 | 23 |
| Lamorna Cove | United Kingdom | 50.0610 | -5.5544 | 30 | 11 | 14 | 25 |
| Pendeen Watch | United Kingdom | 50.1636 | -5.6705 | 60 | 4 | 5 | 9 |
| Porthnanven | United Kingdom | 50.1157 | -5.6996 | 20 | 2 | 3 | 5 |
| Upton Towans | United Kingdom | 50.2100 | -5.3972 | 40 | 2 | 0 | 2 |


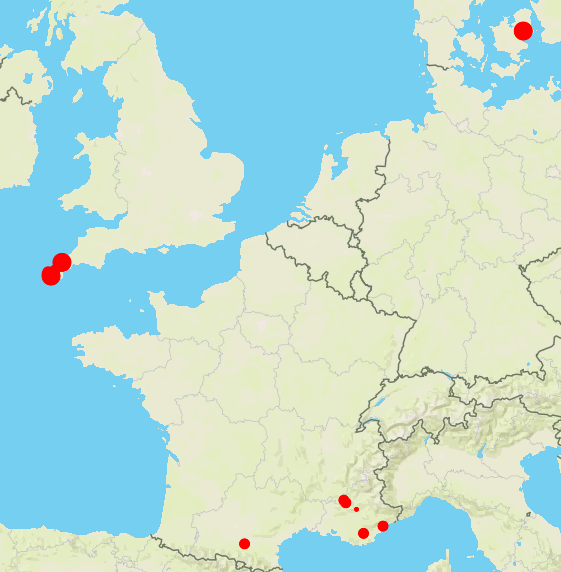

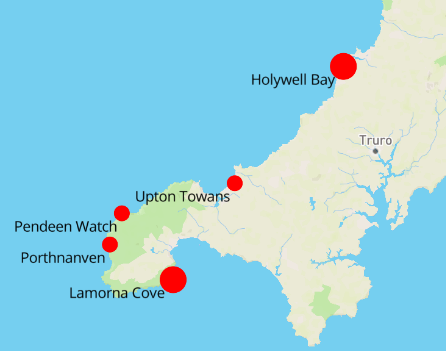


**N° specimens**

1

2 – 10

20+

©OpenStreetMap


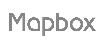


Figure S1b: Map of collection sites; numbers represent specimens photographed.

**S2: Example photographs, showing image analysis approach**


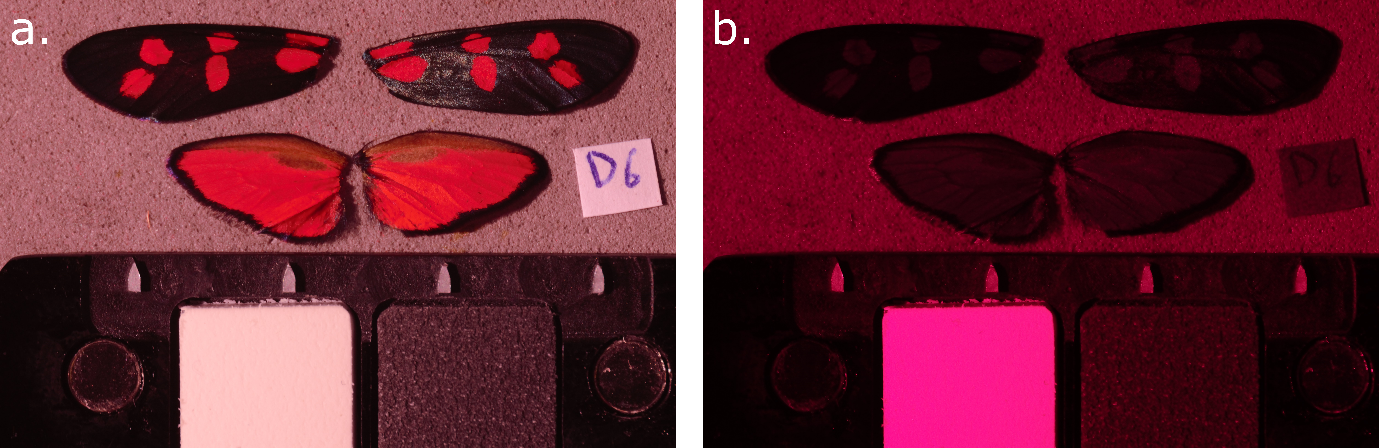


Figure S2: Photographs of one individual’s wings, taken with the UV/infrared blocking filter (a) and the UV pass and IR blocking filter (b). Only the right-hand wings were used for colour measurements.

**S3: Red spot colour in an avian tetrahedral colour space**


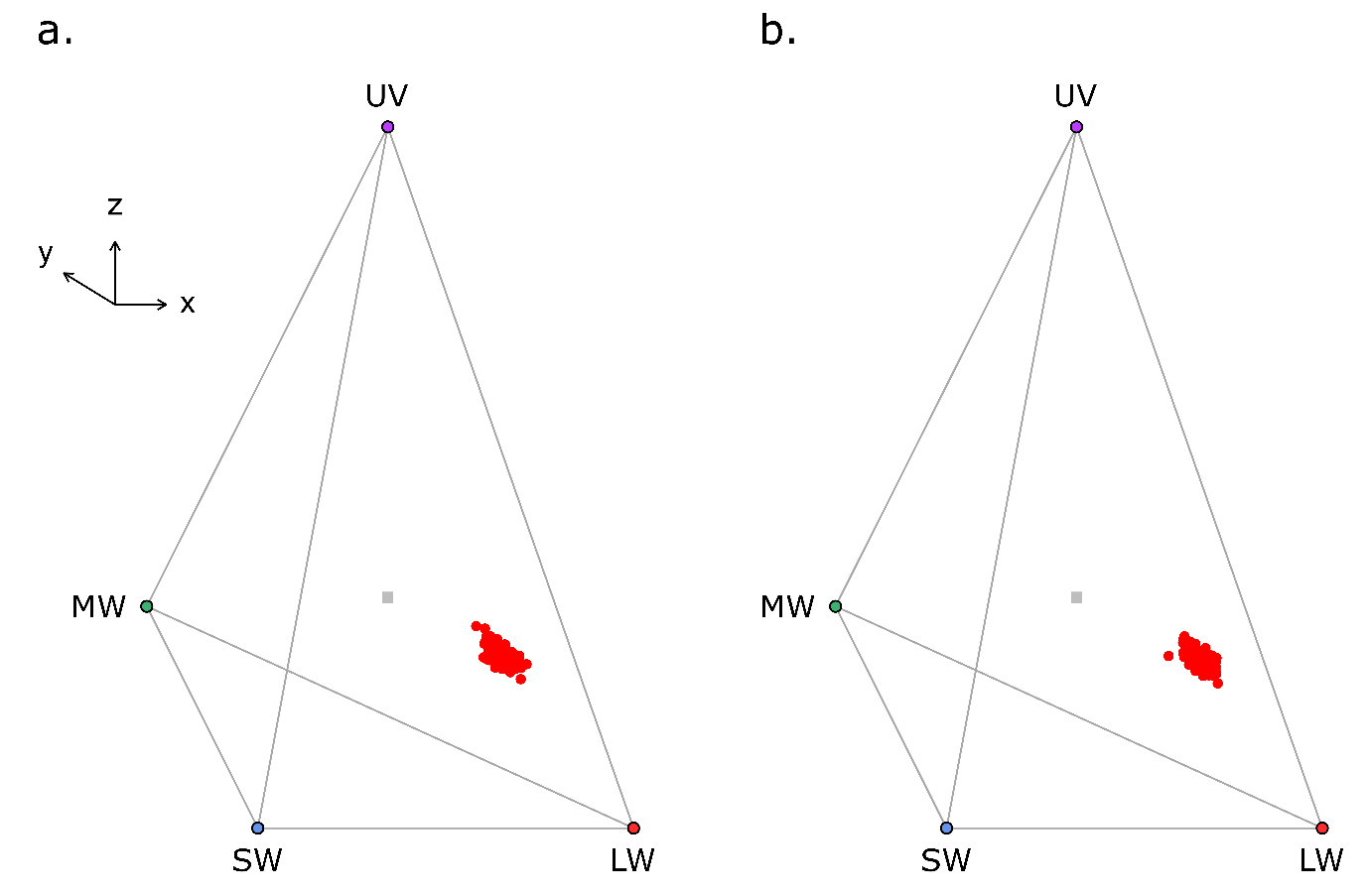
Figure S3: Plots of all individuals’ red area colours in the forewing (a) and hindwing (b) in a tetrahedral colour space, for the UVS visual system. The grey square indicates the achromatic centre of the tetrahedron.

**S4: Full results of model simplification for multiple regression between cyanogenic glucoside concentration, sex and colour metrics in three populations of *Z. filipendulae* (UVS visual system)**

1. Forewings

| **Holywell Bay** |  |  |  | **Holywell Bay** |  |  |  |
| --- | --- | --- | --- | --- | --- | --- | --- |
| **Model including saturation** | | | | **Model including hue** | | | |
| **Factor** | **F** | **df** | **p** | **Factor** | **F** | **df** | **p** |
| Saturation:Sex | 0.11 | 1,11 | 0.75 | Chromatic contrast:Sex | 0.096 | 1,11 | 0.76 |
| Chromatic contrast:Sex | 0.0049 | 1,12 | 0.95 | Hue:Sex | 0.0037 | 1,12 | 0.95 |
| Luminance:Sex | 0.22 | 1,13 | 0.65 | Luminance:Sex | 0.22 | 1,13 | 0.65 |
| Proportion red:Sex | 0.42 | 1,14 | 0.53 | Proportion red:Sex | 0.38 | 1,14 | 0.55 |
| Luminance contrast:Sex | 0.39 | 1,15 | 0.54 | Luminance contrast:Sex | 0.40 | 1,15 | 0.54 |
| Sex | 0.0001 | 1,16 | 0.99 | Sex | 0.0007 | 1,16 | 0.98 |
| Saturation | 0.050 | 1,17 | 0.83 | Hue | 0.13 | 1,17 | 0.72 |
| Luminance contrast | 0.44 | 1,18 | 0.52 | Luminance contrast | 0.44 | 1,18 | 0.52 |
| Proportion red | 1.67 | 1,19 | 0.21 | Proportion red | 1.67 | 1,19 | 0.21 |
| Luminance | 4.36 | 1,20 | *0.050* | Luminance | 4.36 | 1,20 | *0.050* |
| Chromatic contrast | 5.64 | 1,20 | *0.028* | Chromatic contrast | 5.64 | 1,20 | *0.028* |
| **Lamorna Cove** |  |  |  | **Lamorna Cove** |  |  |  |
| **Model including saturation** | | | | **Model including hue** | | | |
| **Factor** | **F** | **df** | **p** | **Factor** | **F** | **df** | **p** |
| Saturation:Sex | 0.027 | 1,11 | 0.87 | Hue:Sex | 0.014 | 1,11 | 0.91 |
| Chromatic contrast:Sex | 0.61 | 1,12 | 0.45 | Chromatic contrast:Sex | 0.53 | 1,12 | 0.48 |
| Luminance:Sex | 1.68 | 1,13 | 0.22 | Luminance:Sex | 1.73 | 1,13 | 0.21 |
| Luminance contrast:Sex | 1.19 | 1,14 | 0.29 | Luminance contrast:Sex | 1.02 | 1,14 | 0.33 |
| Proportion red:Sex | 2.02 | 1,15 | 0.18 | Proportion red:Sex | 1.84 | 1,15 | 0.19 |
| Proportion red | 1.27 | 1,16 | 0.28 | Proportion red | 1.43 | 1,16 | 0.25 |
| Saturation | 1.79 | 1,17 | 0.20 | Hue | 2.03 | 1,17 | 0.17 |
| Chromatic contrast | 0.41 | 1,18 | 0.53 | Chromatic contrast | 0.41 | 1,18 | 0.53 |
| Sex | 1.32 | 1,19 | 0.27 | Sex | 1.32 | 1,19 | 0.27 |
| Luminance contrast | 0.972 | 1,20 | 0.34 | Luminance contrast | 0.97 | 1,20 | 0.34 |
| Luminance | 1.51 | 1,21 | 0.23 | Luminance | 1.51 | 1,21 | 0.23 |
| **Taastrup** |  |  |  | **Taastrup** |  |  |  |
| **Model including saturation** | | | | **Model including hue** | | | |
| **Factor** | **F** | **df** | **p** | **Factor** | **F** | **df** | **p** |
| Chromatic contrast:Sex | 0.0032 | 1,13 | 0.96 | Chromatic contrast:Sex | 0.010 | 1,13 | 0.92 |
| Luminance:Sex | 0.0064 | 1,14 | 0.94 | Luminance:Sex | 0.018 | 1,14 | 0.89 |
| Proportion red:Sex | 0.083 | 1,15 | 0.78 | Proportion red:Sex | 0.098 | 1,15 | 0.76 |
| Luminance contrast:Sex | 0.74 | 1,16 | 0.40 | Luminance contrast:Sex | 0.71 | 1,16 | 0.41 |
| Saturation:Sex | 0.069 | 1,17 | 0.80 | Hue:Sex | 0.079 | 1,17 | 0.78 |
| Chromatic contrast | 0.77 | 1,18 | 0.39 | Chromatic contrast | 0.97 | 1,18 | 0.34 |
| Saturation | 0.14 | 1,19 | 0.71 | Hue | 0.31 | 1,19 | 0.58 |
| Sex | 1.20 | 1,20 | 0.29 | Sex | 1.20 | 1,20 | 0.29 |
| Proportion red | 0.92 | 1,21 | 0.35 | Proportion red | 0.92 | 1,21 | 0.35 |
| Luminance contrast | 3.44 | 1,22 | 0.077 | Luminance contrast | 3.44 | 1,22 | 0.077 |
| Luminance | 6.77 | 1,23 | *0.016* | Luminance | 6.77 | 1,23 | *0.016* |

1. Hindwings

| **Holywell Bay** |  |  |  | **Holywell Bay** |  |  |  |
| --- | --- | --- | --- | --- | --- | --- | --- |
| **Model including saturation** | | | | **Model including hue** | | | |
| **Factor** | **F** | **df** | **p** | **Factor** | **F** | **df** | **p** |
| Saturation:Sex | 0.012 | 1,17 | 0.92 | Hue:Sex | 0.0001 | 1,17 | 0.99 |
| Luminance:Sex | 0.58 | 1,18 | 0.45 | Luminance:Sex | 0.63 | 1,18 | 0.44 |
| Luminance | 0.68 | 1,19 | 0.42 | Luminance | 0.66 | 1,19 | 0.43 |
| Saturation | 0.93 | 1,20 | 0.35 | Hue | 0.77 | 1,20 | 0.39 |
| Sex | 1.52 | 1,21 | 0.23 | Sex | 1.52 | 1,21 | 0.23 |
| **Lamorna Cove** |  |  |  | **Lamorna Cove** |  |  |  |
| **Model including saturation** | | | | **Model including hue** | | | |
| **Factor** | **F** | **df** | **p** | **Factor** | **F** | **df** | **p** |
| Saturation:Sex | 2.78 | 1,17 | 0.11 | Hue:Sex | 4.12 | 1,17 | 0.058 |
| Luminance:Sex | 2.23 | 1,18 | 0.15 | Luminance:Sex | 2.29 | 1,18 | 0.15 |
| Luminance | 0.029 | 1,19 | 0.96 | Hue | 0.023 | 1,19 | 0.88 |
| Saturation | 0.020 | 1,20 | 0.89 | Luminance | 0.016 | 1,20 | 0.90 |
| Sex | 0.47 | 1,21 | 0.50 | Sex | 0.47 | 1,21 | 0.50 |
| **Taastrup** |  |  |  | **Taastrup** |  |  |  |
| **Model including saturation** | | | | **Model including hue** | | | |
| **Factor** | **F** | **df** | **p** | **Factor** | **F** | **df** | **p** |
| Saturation:Sex | 0.0007 | 1,19 | 0.98 | Hue:Sex | 0.0001 | 1,19 | 0.99 |
| Luminance:Sex | 0.18 | 1,20 | 0.68 | Luminance:Sex | 0.17 | 1,20 | 0.68 |
| Saturation | 0.59 | 1,21 | 0.45 | Hue | 0.63 | 1,21 | 0.44 |
| Sex | 0.78 | 1,22 | 0.39 | Sex | 0.78 | 1,22 | 0.39 |
| Luminance | 1.58 | 1,23 | 0.22 | Luminance | 1.58 | 1,23 | 0.22 |

**S5: Toxicity and coloration among populations, for the violet-sensitive (VS) visual system.**

Using the VS model revealed the same results as for the UVS data, although, in addition, forewing chromatic contrast was significantly negatively associated, and luminance contrast positively associated, with higher toxin levels in females. This suggests an even stronger pattern, supporting the UVS results, whereby increased defences are negatively associated with chromatic components of the warning signals (internal chromatic contrast and size of red markings) but positively associated with achromatic visual features (luminance, or perceived lightness, and luminance contrast).

Table S5: Relationship between colour metrics and cyanogenic glucoside concentrations across populations. The same transformations were applied to the data as for the analyses based on the ultraviolet-sensitive (UVS) visual system. Significant results are highlighted in italics. FW=forewing, HW=hindwing.

| **Colour metric** | **Males** | **Females** |
| --- | --- | --- |
| FW luminance | F_1,7_=1.04, p=0.34 | *F_1,9_=15.49, p=0.0034* |
| FW saturation | F_1,7_=0.876, p=0.38 | F_1,9_=3.39, p=0.099 |
| FW hue | F_1,7_=0.86, p=0.38 | F_1,9_=2.87, p=0.12 |
| FW chromatic contrast | F_1,7_=0.57, p=0.48 | *F_1,9_=6.58, p=0.030* |
| FW luminance contrast | F_1,7_=0.67, p=0.44 | *F_1,9_=13.79, p=0.0048* |
| Proportion of red area in FWs | F_1,7_=0.0034, p=0.96 | *F_1,9_=5.25, p=0.048* |
| HW luminance | F_1,7_=0.69, p=0.44 | F_1,9_=1.81, p=0.21 |
| HW saturation | F_1,7_=0.73, p=0.42 | F_1,9_=0.0061, p=0.94 |
| HW hue | F_1,7_=0.75, p=0.41 | F_1,9_=0.0047, p=0.95 |

**S6: Toxicity and coloration within populations, for the violet-sensitive (VS) visual system.**

1. *Relationship between colour and toxicity in Holywell, Lamorna and Taastrup*

Multiple regressions yielded qualitatively identical results to those using the UVS visual model, with one exception: the negative relationship between chromatic contrast and toxicity was no longer significant in the Holywell Bay population.

Table S6a: Results of multiple regressions exploring the relationship between cyanogenic glucoside concentration and colour metrics in the forewings (i) and hindwings (ii). The same transformations were applied to the data as for the analyses based on the ultraviolet-sensitive (UVS) visual system. Results are presented for models including saturation only, as results of models with hue are similar.

i.

| **Factor** | **F** | **df** | **p** |
| --- | --- | --- | --- |
| Holywell Bay |  |  |  |
| Proportion red:Sex | 0.015 | 1,11 | 0.90 |
| Luminance contrast:Sex | 0.11 | 1,12 | 0.75 |
| Saturation:Sex | 0.24 | 1,13 | 0.63 |
| Chromatic contrast:Sex | 0.047 | 1,14 | 0.83 |
| Luminance:Sex | 0.23 | 1,15 | 0.64 |
| Saturation | 0.0001 | 1,16 | 0.99 |
| Proportion red | 0.0003 | 1,17 | 0.99 |
| Chromatic contrast | 0.80 | 1,18 | 0.38 |
| Sex | 2.90 | 1,19 | 0.10 |
| Luminance contrast | 2.94 | 1,20 | 0.10 |
| Luminance | 4.47 | 1,21 | *0.047* |
| Lamorna Cove |  |  |  |
| Saturation:Sex | 0.058 | 1,11 | 0.81 |
| Proportion red:Sex | 0.080 | 1,12 | 0.78 |
| Luminance:Sex | 1.78 | 1,13 | 0.21 |
| Luminance contrast:Sex | 1.44 | 1,14 | 0.25 |
| Chromatic contrast:Sex | 1.34 | 1,15 | 0.26 |
| Luminance contrast | 0.15 | 1,16 | 0.70 |
| Proportion red | 0.65 | 1,17 | 0.43 |
| Saturation | 1.04 | 1,18 | 0.32 |
| Sex | 0.90 | 1,19 | 0.37 |
| Chromatic contrast | 0.84 | 1,20 | 0.37 |
| Luminance | 1.40 | 1,21 | 0.25 |
| Taastrup |  |  |  |
| Chromatic contrast:Sex | 0.0019 | 1,13 | 0.97 |
| Proportion red:Sex | 0.034 | 1,14 | 0.86 |
| Luminance:Sex | 0.092 | 1,15 | 0.76 |
| Luminance contrast:Sex | 0.48 | 1,16 | 0.50 |
| Saturation:Sex | 0.20 | 1,17 | 0.66 |
| Chromatic contrast | 0.14 | 1,18 | 0.72 |
| Saturation | 0.044 | 1,19 | 0.84 |
| Luminance contrast | 0.77 | 1,20 | 0.39 |
| Sex | 1.30 | 1,21 | 0.27 |
| Proportion red | 0.93 | 1,22 | 0.35 |
| Luminance | 6.51 | 1,23 | *0.018* |

ii.

| **Factor** | **F** | **df** | **p** |
| --- | --- | --- | --- |
| Holywell Bay |  |  |  |
| Saturation:Sex | 0.12 | 1,17 | 0.73 |
| Luminance:Sex | 0.23 | 1,18 | 0.64 |
| Luminance | 0.68 | 1,19 | 0.42 |
| Sex | 1.31 | 1,20 | 0.27 |
| Saturation | 1.96 | 1,21 | 0.18 |
| Lamorna Cove |  |  |  |
| Saturation:Sex | 3.12 | 1,17 | 0.095 |
| Luminance:Sex | 1.85 | 1,18 | 0.19 |
| Saturation | 0.0036 | 1,19 | 0.95 |
| Luminance | 0.12 | 1,20 | 0.73 |
| Sex | 0.47 | 1,21 | 0.50 |
| Taastrup |  |  |  |
| Saturation:Sex | 0.061 | 1,19 | 0.81 |
| Luminance:Sex | 0.18 | 1,20 | 0.68 |
| Saturation | 0.34 | 1,21 | 0.57 |
| Sex | 0.91 | 1,22 | 0.35 |
| Luminance | 1.46 | 1.23 | 0.24 |

1. *Sex differences in coloration*

Population and sex differences in colour metrics were very similar between visual models. Nevertheless, with the VS visual model, some population-level differences in forewing luminance, saturation and hue became significant: markings in the Taastrup population were significantly lighter (p_Holywell-Lamorna_=0.52, p_Holywell-Taastrup_=0.36, p_Lamorna-Taastrup_=0.037), less saturated (p_Holywell-Lamorna_=0.79, p_Holywell-Taastrup_=0.19, p_Lamorna-Taastrup_=0.032) and with lower hue values (p_Holywell-Lamorna_=0.86, p_Holywell-Taastrup_=0.16, p_Lamorna-Taastrup_=0.034) than those from Lamorna Cove. This suggests that these two populations differ more, whether chromatically or achromatically, to a VS visual model. However, there were no significant differences in luminance contrast with a VS model, whereas, under a UVS model, Lamorna Cove moths appeared to display lower luminance contrast in their hindwings than moths of other populations. As there is no significant difference in cyanogenic glucoside levels between the Taastrup and Lamorna Cove populations, these population-level differences in coloration, whether recorded the VS or UVS visual systems, are unlikely to constitute a useful signal of variation in toxicity for predators.

Table S6b: Results of linear models testing for sex and population-level differences in colour metrics across Holywell Bay, Lamorna Cove and Taastrup. Unlike the UVS data, luminance contrast was not logit-transformed.

| **Factor** | **F** | **df** | **p** | **F** | **df** | **p** | **F** | **df** | **p** |
| --- | --- | --- | --- | --- | --- | --- | --- | --- | --- |
|  | FW luminance | | | FW saturation | | | FW hue | | |
| Sex:Population | 1.10 | 2,67 | 0.34 | 1.22 | 2,67 | 0.30 | 1.39 | 2,67 | 0.26 |
| Population | 3.68 | 2,69 | *0.030* | 3.46 | 2,69 | *0.037* | 3.46 | 2,69 | *0.037* |
| Sex | 1.27 | 1,69 | 0.26 | 4.37 | 1,69 | *0.040* | 4.38 | 1,69 | *0.040* |
|  | Proportion red | | | Chromatic contrast | | | Luminance contrast | | |
| Sex:Population | 1.51 | 2,67 | 0.23 | 1.36 | 2,67 | 0.26 | 0.051 | 2,67 | 0.95 |
| Population | 2.28 | 2,69 | 0.11 | 4.25 | 2,69 | *0.018* | 1.15 | 2,69 | 0.32 |
| Sex | 17.77 | 1,71 | *<0.001* | 19.29 | 1,69 | *<0.001* | 0.29 | 1,71 | 0.65 |
|  | HW luminance | | | HW saturation | | | HW hue | | |
| Sex:Population | 0.44 | 2,67 | 0.64 | 1.41 | 2,67 | 0.25 | 1.51 | 2,67 | 0.23 |
| Population | 7.79 | 2,69 | *<0.001* | 2.41 | 2,69 | 0.097 | 2.61 | 2,69 | 0.080 |
| Sex | 16.06 | 1,69 | *<0.001* | 30.38 | 1,71 | *<0.001* | 7.31 | 1,71 | *<0.01* |

1. *Contrast between wing and natural background colours*

Results were qualitatively identical to those based on the UVS model for chromatic contrast to plants, and very similar for luminance contrast. However, population-level differences in luminance contrast became significant with the VS model:markings in Taastrup were less contrasting than those in Lamorna Cove, although this once again is unrelated to differences in toxin levels..

Table S6c: Results of mixed models testing differences in contrast between forewing markings in Holywell Bay, Lamorna Cove and Taastrup, and natural backgrounds. Lc=*Lotus corniculatus*, Ka=*Knautia arvensis.*

| **Factor** | **χ^2^** | **df** | **p** | **Tukey’s post-hoc tests** |
| --- | --- | --- | --- | --- |
| Chromatic contrast |  |  |  |  |
| Sex:Population | 3.21 | 2 | 0.20 | - |
| Sex | 4.88 | 1 | *0.027* | - |
| Population | 15.57 | 2 | *<0.001* | p_Holywell-Lamorna_=0.32, p_Holywell-Taastrup_=0.048, *p_Lamorna-Taastrup_<0.001* |
| Plant type | 638.0 | 2 | *<0.001* | *p_Lc leaves-Lc flowers_ <0.001, p_Lc leaves-Ka flowers_<0.001, p_Lc flowers-Ka flowers_ <0.001* |
| Luminance contrast |  |  |  |  |
| Sex:Population | 2.36 | 2 | 0.31 | - |
| Sex | 1.27 | 1 | 0.26 | - |
| Population | 6.32 | 2 | *0.043* | p_Holywell-Lamorna_=0.53, p_Holywell-Taastrup_=0.35, *p_Lamorna-Taastrup_=0.032* |
| Plant type | 852.92 | 2 | *<0.001* | *p_Lc leaves-Lc flowers_ <0.001, p_Lc leaves-Ka flowers_<0.001, p_Lc flowers-Ka flowers_ <0.001* |
